# Similarities and Differences: A Comparative Review of the Molecular Mechanisms and Effectors of NAFLD and AFLD

**DOI:** 10.3389/fphys.2021.710285

**Published:** 2021-07-30

**Authors:** Pengyi Zhang, Weiya Wang, Min Mao, Ruolin Gao, Wenting Shi, Dongmei Li, Richard Calderone, Bo Sui, Xuewen Tian, Xiangjing Meng

**Affiliations:** ^1^School of Sports and Health, Shandong Sport University, Jinan, China; ^2^Shandong Academy of Pharmaceutical Science, Jinan, China; ^3^Department of Allied Health, University of North Carolina at Chapel Hill, Chapel Hill, NC, United States; ^4^Department of Microbiology and Immunology, Georgetown University Medical Center, Washington, DC, United States

**Keywords:** NAFLD, AFLD, signaling pathway, clinical trials, effectors, similarities and differences

## Abstract

Non-alcoholic fatty liver disease (NAFLD) and alcoholic fatty liver disease (AFLD) are the most prevalent metabolic liver diseases globally. Due to the complex pathogenic mechanisms of NAFLD and AFLD, no specific drugs were approved at present. Lipid accumulation, oxidative stress, insulin resistance, inflammation, and dietary habits are all closely related to the pathogenesis of NAFLD and AFLD. However, the mechanism that promotes disease progression has not been fully elucidated. Meanwhile, the gut microbiota and their metabolites also play an important role in the pathogenesis and development of NAFLD and AFLD. This article comparatively reviewed the shared and specific signaling pathways, clinical trials, and potential intervention effectors of NAFLD and AFLD, revealing their similarities and differences. By comparing the shared and specific molecular regulatory mechanisms, this paper provides mutual reference strategies for preventing and treating NAFLD, AFLD, and related metabolic diseases. Furthermore, it provides enlightenment for discovering novel therapies of safe and effective drugs targeting the metabolic liver disease.

Non-alcoholic fatty liver disease (NAFLD) and alcoholic fatty liver disease (AFLD) are the main metabolic liver diseases that are prevalent worldwide. Metabolic liver disease seriously affects human health and quality of life, leading to major public health problems and huge medical burdens (Estes et al., 2018; Sarin et al., 2020). NAFLD is associated with metabolic syndromes (MS) such as obesity, type 2 diabetes mellitus (T2DM), and dyslipidemia (Younossi and Henry, 2016; Zhang X. et al., 2018). The liver histology of NAFLD showed macrovesicular steatosis, mild lobular inflammation, and non-alcoholic ballooned hepatocytes (Loomba et al., 2021). NAFLD affects ~25% of the global adult population (Younossi et al., 2019). AFLD is associated with excessive alcohol consumption and accounts for 47.9% of cirrhosis deaths worldwide. AFLD has become the most common indication for liver transplantation in the United States (Rehm et al., 2013; Johnston et al., 2020). Lipid accumulation, oxidative stress, insulin resistance (IR), inflammation, and dietary habits are all closely related to the pathogenesis of NAFLD and AFLD. However, the mechanisms that drive disease progression have not been fully elucidated. Due to the complex pathogenic mechanisms of NAFLD and AFLD, there are no approved specific drugs at present. The clinical treatment mainly controls the disease progression and risk factors by lipid-regulating and anti-inflammatory drugs, antioxidants, weight loss, and hypoglycemia. The prevention and treatment strategies for these two diseases are very limited in type and efficacy. Therefore, this article systematically reviewed and comparatively analyzed the shared and specific molecular mechanisms and intervention effectors of NAFLD and AFLD, to fully understand their pathogenic mechanisms and provide ideas for screening and discovering novel prevention strategies and therapeutic targets of metabolic fatty liver disease.

## Introduction

NAFLD and AFLD are public health issues worldwide. These two diseases have a similar pathological spectrum, from simple hepatic steatosis to steatohepatitis with or without fibrosis, to cirrhosis and liver cancer. NAFLD is one of the main causes of chronic hepatitis, and it is also the only cause of a steady increase of the global liver disease incidence over time, especially in developing countries such as the Middle East or South America (Younossi et al., 2018). It is estimated that by 2030, the prevalence of the end-stage liver disease will increase by 2–3 times in Western countries and several Asian countries (Estes et al., 2017, 2018). The pathogenesis of NAFLD involves several risk factors, including obesity, IR, hyperglycemia, diabetes, hypertension, dyslipidemia, aging, and decreased physical activity, among which obesity is the most important risk factor for the development of NAFLD (Schwimmer, 2007). The prevalence of NAFLD in obese people can reach 57.5–74%, which is 4.6 times that of people with normal weight (Angulo and Lindor, 2002). The risk of NAFLD and non-alcoholic steatohepatitis (NASH) is also increased due to the imbalance of gut microbiota, increased intestinal permeability, and translocation of intestinal microbes (Chu et al., 2018). Therefore, the changes of gut microbiota diversity and abundance may be another important factor causing metabolic syndrome (Bashiardes et al., 2016). The elevated fasting plasma ethanol level was suggested to be critical for the development of NAFLD. On the one hand, it is due to the increased synthesis of endogenous alcohol in the intestine. It is reported that *Klebsiella pneumoniae* was found in a considerable proportion of fatty liver patients, which can produce a large amount of alcohol in their intestines. The endogenous alcohol produced by these bacteria is an important inducer of NAFLD (Shellito et al., 2001). On the other hand, in a study of children with early signs of NAFLD, the fasting plasma ethanol levels were positively associated with measures of IR in children with NAFLD compared with the control group. *In vivo* experiments showed that ADH activity was significantly lower in liver of ob/ob mice. Thus, the impaired insulin-dependent ADH activity in liver led to increased plasma ethanol levels in NAFLD patients, rather than increased endogenous ethanol synthesis (Engstler et al., 2016). AFLD is a fatty liver disease with fat accumulation and inflammation caused by excessive alcohol consumption, affecting more than two million people in the United States. The prevalence of AFLD is not only affected by alcohol abuse, gut microbiota, immunity, gender, genetic defects, and other environmental factors also play an important role in the progression of AFLD patients (Tilg and Mathurin, 2016).

## Signaling Pathway of NAFLD and AFLD

The pathogenesis of NAFLD is regulated by many molecular mechanisms (Friedman et al., 2018), which is usually caused by multiple factors such as lipid metabolic dysfunction, oxidative stress, IR, and inflammation (Hardy and Mann, 2016; Eslam et al., 2018). In AFLD, excessive alcohol consumption produces a large amount of acetaldehyde, which accumulates when acetaldehyde dehydrogenase (ALDH) is relatively deficient. Excess acetaldehyde will produce more reactive oxygen species (ROS), leading to oxidative stress and hepatocyte damage. At the same time, it can form various proteins and DNA complexes, which act as antigens to activate adaptive immunity and increase inflammation (Lackner et al., 2016; Xu et al., 2017; Anty and Gual, 2019). Ethanol can also cause dyslipidemia and lipid accumulation to form lipid droplets (LDs). Excessive LDs are susceptible to the attack of ROS produced by ethanol metabolism, thereby synergistically aggravating oxidative stress and liver damage. Through systematic review and comparative analysis of the signaling pathways of NAFLD and AFLD, the common and specific signal pathways of both are revealed, and novel therapeutic strategies are provided for the treatment of NAFLD and AFLD.

## NAFLD Signaling Pathway

This paper mainly introduces six signaling pathways that regulate NAFLD: ① Nrf2/FXR/LXRα/RXR/SREBP-1c signaling pathway is an important pathway that maintains the intracellular redox balance and regulates lipid metabolism. NF-E2-related factor 2 (Nrf2) is an important upstream transcription factor that regulates oxidative stress, and it is also a central regulator of intracellular redox homeostasis. The regulation of its expression is an important antioxidant defense mechanism of the body (Dodson et al., 2019). Nrf2 recruits p300 to promote the deacetylation of farnesoid X receptor (FXR) in primary hepatocytes isolated from wild-type male C57/BL6 mice. Then small heteredodimer partner (SHP) was induced by deacetylated FXR to inhibit liver X receptor α (LXRα)-dependent gene transcription. In human steatosis, Nrf2, FXR, and SHP have negative regulatory effects on LXRα and sterol regulatory element binding protein-1c (SREBP-1c). LXRα and its target SREBP-1c transcriptionally regulate fatty acid synthesis. The activation of Nrf2 suppresses the transcription of LXRα, SREBP-1c, and LXRα-dependent hepatic steatosis through FXR activation and FXR-mediated induction of SHP (Kay et al., 2011; Fan et al., 2021). LXRα and Retinoid X receptor (RXR) can form a heterodimer, which further activates SREBP-1c. SREBP-1c is the main transcription factor regulating fatty acid synthesis. It participates in the regulation of lipid and cholesterol homeostasis by positively regulating the transcription of acetyl-CoA carboxylase (ACC) and fatty acid synthase (FAS) (Chávez-Talavera et al., 2017; Hiebl et al., 2018). On the other hand, tissue-restricted FXR agonists can improve insulin sensitivity and reduce hepatic steatosis in HFD mice and non-diabetic NAFLD patients (Carino et al., 2017; Traussnigg et al., 2021). FXR is a ligand-activated transcriptional factor. The release of bile acids (BAs) during meals selectively activates intestinal FXR. The intestinal-restricted FXR agonist fexaramine (Fex) induces intestinal fibroblast growth factor 15 (FGF15), which changes the composition of BAs but does not activate FXR target genes in the liver. In HFD mice treated with Fex (100 mg kg^−1^ d^−1^ p.o. for 5 weeks), Fex improves diet-induced body weight gain, systemic inflammation, and the lipid metabolism of liver by activating FXR (Fang et al., 2015). The activation of FXR is beneficial for the homeostasis of lipid and glucose, energy metabolism, hepatic steatosis (Gai et al., 2018), cell stress, and the intake of intestinal Bas (Han et al., 2018). FXR is also an important gene that regulates lipid metabolism in AFLD. The intestine-specific FXR knockout (FXRint^−/−^) mice were more likely to cause hepatic steatosis and inflammation under alcohol induction (Huang M. et al., 2020). However, the results are not completely consistent between animal models and human research. The changes in the diversity, abundance and metabolites of gut microbiota are one potential reason worth investigating (Adorini et al., 2012; Arab et al., 2017; McIlvride et al., 2019).

② PI3K/AKT/SREBP-1c signaling pathway is an important pathway for regulating lipid metabolism, which can reduce mitochondrial oxidative stress, inhibit adipogenesis, delay excessive lipid deposition, and alleviate hepatic steatosis (Abood et al., 2018; Liao et al., 2018; Pan et al., 2018). The PI3K/AKT signaling pathway even reprograms cell carcinogenesis by regulating lipid and protein biosynthesis in mouse liver (Palian et al., 2014). Poly ADP-ribose polymerase 1(PARP-1) expressed in the nucleus is a key regulatory enzyme for DNA repair and chromatin structure maintenance. Under pathological stimulation, PARP-1 overactivation may cause cell damage, accompanied by energy depletion, mitochondrial dysfunction, and β-oxidation disruption in mouse leukemia cells (Cantó and Auwerx, 2011). The PI3K/AKT signaling pathway can be activated by the inhibition of PARP-1. AKT is an important downstream effector molecule of PI3K. Puerarin can activate this signaling pathway to down-regulate the lipid synthesis-related gene SREBP-1c, thereby reducing lipid biosynthesis and achieving the effect of improving fatty liver disease in C57BL/6J mice fed with a high-fat high-sucrose (HFHS) diet (Wang et al., 2019). PARP-1 not only directly affects the expression of liver adipose gene through transcriptional regulation, but also indirectly regulates the energy metabolism by affecting liver nicotinamide adenine dinucleotide (NAD^+^) consumption and Sirtuin 1 (SIRT1) activity. The inhibition of PARP-1 can increase NAD^+^ content and SIRT1 activity, enhance lipid metabolism, and improve hepatic steatosis in hepatocytes under oxidative stress and mice fed a high-fat diet (Kraus and Hottiger, 2013). ③ Amp-activated protein kinase (AMPK)/Sarcoendoplasmic reticulum Ca^2+^-ATPase 2b (SERCA2b) pathway mediates suppression of endoplasmic reticulum (ER) stress to improve hepatic steatosis. In the case of hyperlipidemia, the reduction of ER stress in hepatocytes is the most common therapy to inhibit lipid accumulation. When AMPK is activated by maresin 1, it positively regulates the activation of SERCA2B. SERCA2B re-uptake Ca^2+^ from the cytoplasm into the ER cavity, alleviating the ER stress of hepatocytes under hyperlipidemia and maintaining ER homeostasis, thereby inhibiting lipid accumulation (Jung et al., 2018). Conversely, the inhibition of AMPKα1 promoted the development of NAFLD in HFD mice through increasing adipocyte-mediated CD36-containing exosomes release. The CD36-containing exosomes induced lipid accumulation and inflammation in hepatocytes, which could be reversed by metformin (Yan et al., 2021). ④ LILRB4/SHP1/TRAF6/NF-κB/MAPK signaling pathway can improve the development of NAFLD and related metabolic complications. Leukocyte immunoglobulin-like receptor (LILRB4) is the major receptor in the immunoglobulin superfamily, mainly located on the membrane of immune cells (Katz, 2007). The activation of LILRB4 contributes to the improvement of inflammation. LILRB4 negatively regulates protein tyrosine phosphatase (SHP1), inhibits the ubiquitination of TNF receptor associated factor 6 (TRAF6), and prevents the activation of nuclear factor kappa-B (NF-κB) and mitogen-activated protein kinase (MAPK) signaling pathways. Thus, IR, glucose metabolism imbalance, liver lipid accumulation, hepatic steatosis, and systemic inflammation in mice fed a high-fat diet could be reversed (Lu et al., 2018). ⑤ The lipid metabolism can be improved by inhibiting TXNIP/NLRP3 signaling pathway. Thioredoxin intervening protein (TXNIP), as an important mediator of redox homeostasis, was overexpressed in the liver of streptozotocin-induced diabetic rats. The high expression of TXNIP is closely related to the occurrence of oxidative stress and inflammation in the liver. The reduction of its expression can effectively inhibit the production of intracellular ROS (Zhou and Chng, 2013). NOD-like receptor protein 3 (NLRP3) inflammasome is a multi-protein complex composed of intracellular innate immune receptor NLRP3, adaptor protein ASC, and protease caspase-1, which can induce the maturation and secretion of pro-inflammatory factors IL-1β and IL-18, and promote the inflammatory response (Jiang H. et al., 2017). Inhibiting the overexpression of TXNIP can inhibit the activation of NLRP3 inflammasomes, reduce the secretion of pro-inflammatory factors, and then regulate the expression of lipid metabolism genes in rat BRL-3A hepatocytes treated with sodium palmitate (SP) (Xiao et al., 2016). NLRP3 inflammasome is crucial for the initiation of hepatic inflammatory response and the progression from hepatic steatosis to NASH. The inhibition of its endogenous activation can delay the progression of NAFLD in female Sprague-Dawley rat fed a high-fat diet (Henao-Mejia et al., 2012). ⑥ TAZ/Ihh signaling pathway: The transcription expression of hippo transcription regulator-Tafazzin (TAZ) and its downstream target hepatic stellate cell fibrosis gene activator-Indian Hedgehog (Ihh) is significantly increased in the livers of NASH patients. This phenomenon has also been verified in mouse models. TAZ promotes hepatocyte fibrosis by inducing Ihh in NASH mice. Silencing TAZ can prevent and reverse NASH, especially liver fibrosis, but not hepatic steatosis (Wang X. et al., 2016). p62/Sqstm1 was another TAZ target gene down-stream, which played an important role in inflammatory and hepatocyte injury in NASH mice. TAZ was able to regulate the expression of p62/Sqstm1 to alleviate NASH both *in vitro* and *in vivo* (Yang et al., 2021).

In addition, plasminogen activator inhibitor-1 (PAI-1) is a serine protease inhibitor and a major inhibitor of the endogenous fibrinolytic system. The expression of plasma PAI-1 was increased in people with obesity, IR, and metabolic syndromes (Cesari et al., 2010). The plasma PAI-1 level had a positive correlation with hepatic steatosis, lobular inflammation, balloonosis, and fibrosis in children with NAFLD. PAI-1 is also significantly correlated with blood lipids and IR index. PAI-1 can be used as a potential diagnostic marker and drug target for determining the progression of NAFLD (Jin et al., 2018). The animal models of NAFLD induced by HFD had very similar pathological changes to human diseases, such as obesity, dyslipidemia and IR, but only led to very little fibrosis. In fact, the therapeutic effects obtained in animal models of NAFLD were not completely consistent with human clinical trials (Tarantino et al., 2019). Nevertheless, *in vitro* and *in vivo* experiments contribute to discover and understand the multiple molecular mechanisms of NAFLD, and provide potential candidate strategies for human trials, clinical treatments and new drug screening.

## AFLD Signaling Pathway

Ethanol can lead to the disorder of liver lipid metabolism and the production of inflammatory cytokines. The study of the pathogenesis and potential signal transduction pathways of AFLD has important clinical research value in preventing hepatic steatosis patients from developing more severe steatohepatitis, liver fibrosis/cirrhosis, and eventually hepatocellular carcinoma (HCC). For this reason, our paper focuses on six signaling pathways that regulate AFLD: ① SIRT1/AMPK/Lipin-1 signaling pathway: The signal transduction mechanism of AFLD involves a upstream signaling regulation system SIRT1/AMPK, with Lipin-1 as a downstream key regulator. Lipin-1 is a phospholipid acid phosphatase (PAP) that plays a dual role in promoting triglyceride (TG) synthesis during lipid metabolism and acting as a transcriptional auxiliary regulator in the nucleus. Firstly, ethanol can increase the gene and protein expression of Lipin-1, induce Lipin-1-PAP activity and nucleocytoplasmic shuttling, inhibit Lipin-1-mediated fatty acid oxidation and VLDL-TG secretion in the liver, disrupt SIRT1-SFRS10 axis to affect Lpin1 alternative splicing, interfere with Lipin-1/NF-κB/NFATc4 axis, induce the production of pro-inflammatory cytokines, and finally lead to the development of AFLD in cultured hepatocytes and in mouse livers with alcoholic hepatitis (You et al., 2017). Secondly, ethanol seriously impaired liver peroxisome proliferator-activated receptor-γ coactivator-1α (PGC-1α)/peroxisome proliferator-activated receptor α (PPARα) signal transduction and aggravated steatohepatitis in several mouse models of AFLD. Lipin-1 was transferred to the nucleus, where it co-activated PGC-1α and PPARα, enhancing the expression of mitochondrial genes involved in fatty acid oxidation. Consequently, hepatic steatosis induced by ethanol exposure was prevented (Fischer et al., 2003; Nakajima et al., 2004). In addition, ethanol-induced endoplasmic reticulum (ER) stress and SIRT1-AMPK signaling system damage caused the activation of mTORC1-nuclear lipin-1-SREBP-1 signaling pathway in AFLD mice (You et al., 2004). In hepatocytes, nuclear Lipin-1 is the key component of mTORC1-SREBP-1 pathway (Bakan and Laplante, 2012). Lipin-1 is a nucleocytoplasmic shuttling protein. The nuclear-located Lipin-1 depends on its PAP activity to inhibit the function of SREBP-1, thus reducing diet-induced hepatic steatosis. A better understanding of the regulatory mechanism of Lipin-1 under the action of ethanol will facilitate the development of novel pharmacological or nutritional therapies for the treatment of alcoholic steatosis/steatohepatitis patients (Peterson et al., 2011). ② PI3K/AKT/Nrf2/PPARγ signaling pathway: The excessive consumption of alcohol produces large amounts of ROS. The oxidative stress induced by ROS down-regulates the expression of PI3K and AKT, thus inhibiting its downstream target Nrf2 in male BALB/c mice (Liu et al., 2019; Wang et al., 2021). NRF2 and peroxisome proliferator-activated receptor γ (PPARγ) are two signaling pathways that regulate each other with positive feedback (Reddy and Standiford, 2010). Under oxidative stress, the expression of PPAR γ in Nrf2 knockout mice decreased significantly (Lee, 2017). PPARγ alleviates inflammation and oxidative stress by inhibiting the expression of NLRP3 in AFLD mice (Haneklaus and O'Neill, 2015; Hughes and O'Neill, 2018; Meng et al., 2019). ③ p62/Nrf2/KEAP1 signaling pathway: NRF2 has become a key site for the treatment of AFLD. It plays a role in the regulation of heme oxygenase 1 (HO-1) and glutathione (GSH) (Gyamfi and Wan, 2010; Li et al., 2015). The activated Nrf2 enters the nucleus, binds to the antioxidant element ARE, and then activates the expression of HO-1 and antioxidant proteases to resist internal and external stimuli in AML12 hepatocyte cells (Ge et al., 2017; Jiang L. et al., 2017). Nrf2 mediates the expression of p62 gene induced by oxidative stress, and p62 protein also contributes to the activation of Nrf2, forming a positive feedback loop (Jain et al., 2010). ARE, the antioxidant component of p62 promoter, induces oxidative stress through NRF2. Also, p62 connects to the Kelch-repeat domain of Kelch-like ECH-associated protein 1 (KEAP1) in the cytoplasm through the Keap1 interaction region (KIR), blocking the binding of KEAP1 and NRF2, leading to ubiquitination and degradation of transcription factors. The interaction between KEAP1 and p62 leads to the accumulation and autophagy degradation of endogenous KEAP1, which regulates the activation of Nrf2 (Qiu et al., 2017; Zhao et al., 2021). ④ STING-IRF3-Bax signaling pathway induces inflammation, ER stress, and apoptosis to cause hepatocyte injury and dysfunction. AFLD could be improved by inhibiting the expression of this pathway. Innate immunity is one of the driving factors of AFLD. As a mediator of innate immune signaling pathways, the stimulator of interferon genes (STING) connects the upstream DNA sensors with the downstream interferon regulatory factor 3 (IRF3). When ER stress was induced by ethanol, it caused the binding of STING, ER adapter, and IRF3. The phosphorylation of IRF3 activated B-cell lymphoma 2 (Bcl2)-associated X protein (Bax), leading to the apoptosis of hepatocytes in mice fed 4-week ethanol (Ishikawa and Barber, 2008; Petrasek et al., 2013). ⑤ C3/CYP2E1/Gly-tRF/SIRT1 complement regulation system: The C3 complement activation product C3a regulates CYP2E1 (a member of the cytochrome P450 mixed function oxidase system) to restore the expression of glycine transfer (t) RNA-derived fragments (Gly-tRF). The expression of Gly-tRF was increased in ALD patients to down-regulate the expression of downstream SIRT1, which promoted adipogenesis and inhibit fatty acid β oxidation. The complement regulation system plays a key role in the development of hepatic steatosis (Zhong et al., 2019). ⑥ LRP6/Wnt/β-catenin/CYP2E1 signaling pathway: CYP2E1 produces ROS under the action of ethanol, which leads to lipid peroxidation and DNA damage in hepatocytes of rats fed ethanol for 1 month (French, 2013). Low density lipoprotein receptor-related protein6 (LRP6) is a key protein that regulates the Wnt/β-catenin signaling pathway (Go, 2015). LRP6 can regulate the expression of CYP2E1 through the Wnt/β-catenin signaling pathway studied in liver-specific Lrp5/6 KO (Lrp-LKO) and conditional Wntless (Wls) KO mice (Yang et al., 2014), thereby affecting the susceptibility of individuals to alcoholic hepatic injury, which were investigated in AFLD patients. The results were verified in LRP6^(+/−)^ mice fed with 2.4 or 4 g/kg ethanol every 7 days for 28 days (Xu et al., 2019).

In addition, p66Shc, a subtype of the shcA adaptor protein family, is an inducible oxidoreductase that regulates the production of ROS and H_2_O_2_. In the presence of ethanol, p66Shc can cause oxidative damage to hepatocytes (Giorgio et al., 2005; Berniakovich et al., 2008). The bioinformatics analysis indicated that p66Shc was a potential target gene of miR-219a-5p. In *in vivo* and *in vitro* experiments, miR-219a-5p can directly inhibit the expression of p66Shc to improve oxidative stress in Male Sprague-Dawley rats and AML12 hepatocyte cells (Fu et al., 2019).

## Shared Signaling Pathway of NAFLD and AFLD

As an upstream signaling system, the SIRT1/AMPK signaling pathway is responsible for regulating hepatic lipid metabolism. SIRT1/AMPK regulates each other and shares many molecular mechanisms, playing an important role in lipid metabolism in mice with streptozotocin/nicotinamide-induced diabetes and db/db mice fed on a high-fat diet (Chen et al., 2012). The deacetylation activity of SIRT1 is driven by NAD^+^ levels. A significant increase in the ratio of NAD^+^/NADH activates the expression of SIRT1, and then SIRT1 activates the phosphorylation of AMPK. AMPK is a sensor of the cellular energy state, which is activated by AMP and inhibited by ATP. AMPK is also activated by phosphorylation of the liver kinase B1 (LKB1). An increase in the ratio of NAD^+^/NADH, results in the phosphorylation of LKB1 and AMPK which can activate the SIRT1/AMPK signaling pathway, inhibit adipogenesis and promote lipid oxidation, and reduce NAFLD symptoms and IR in HFD mice (Park et al., 2016). Insulin was able to activate SREBP-1c directly and increase the activity of mTORC1 to regulate SREBP-1c indirectly. In addition, SREBP-1c also regulated hepatic lipid synthesis independently of insulin (Loomba et al., 2021). On the other hand, the activation of SREBP-1 by ethanol can also be partially inhibited by AMPK. In the process of fatty acid metabolism, the phosphorylation of AMPK inhibits the activation of SREBP-1 and positively regulates the expression of PPARα, thereby regulating lipid metabolism homeostasis and improving AFLD in rat hepatic cells and in the livers of mice fed with ethanol (Zhou et al., 2001; You et al., 2004; Berger et al., 2005). Activated PPARα up-regulates the transcription of carnitine palmitoyl transferase 1 (CPT-1) and acyl coenzyme A oxidase 1 (ACOX1). As an inhibitor of fatty acid oxidation in mitochondria, CPT-1 can regulate the uptake of fatty acids. ACOX1 is a key enzyme in the oxidation of peroxisomal β. The down-regulation of this gene leads to the dysfunction of long-chain fatty acids oxidation and hepatic steatosis in HepG2 cells (Zeng et al., 2016). In HFD-induced NAFLD mice and oleic acid-induced FL83B cells, fisetin (a natural flavonoid derived from fruits and vegetables), significantly increased phosphorylation of AMPKα, production of Sirt1, and β-oxidation in hepatocytes, decreased lipid accumulation. Fisetin improved NAFLD by regulating the SIRT1/AMPK pathway and fatty acid β oxidation (Liou et al., 2018).

Also, SIRT1-AMPK is a central target of ethanol in the liver, regulating the expression of adiponectin (ADPN), a key factor in AFLD. ADPN has a protective effect on AFLD. In AFLD, the expression of ADPN and its receptors are decreased, leading to damage to the hepatic adiponectin signaling pathway in ethanol-fed mice (You and Rogers, 2009). In addition, ethanol exposure also causes ER stress, and SIRT1-AMPK is driven to regulate the expression of Lipin-1. The increase in Lipin-1 transcription and expression is related to the oxidation of fatty acid, the secretion of VLDL-TG, and the reduction of pro-inflammatory factors and lipid accumulation in cultured hepatocyte cells and in the livers of rodents (You et al., 2017). SIRT1/AMPK/SREBP-1c is a key pathway for regulating lipid metabolism in both NAFLD and AFLD (Chen et al., 2012; Quan et al., 2013). SREBP-1c is activated by ER stress and positively regulates the transcription of stearoyl-CoA desaturase 1 (SCD1), FAS, acetyl-CoA carboxylase (ACC), and ATP citrate lyase (Acly), which directly catalyze adipogenesis (Ferré and Foufelle, 2010; Fortin et al., 2017). Acly is responsible for the synthesis of Acetyl-CoA, and ACC converts Acetyl-CoA to Malonyl-CoA through carboxylation. FAS is responsible for the synthesis of long-chain fatty acids from Acetyl-CoA and Malonyl-CoA, while SCD1 is responsible for the synthesis of unsaturated fatty acids (Chirala et al., 2001; Huang et al., 2013). SCD1 is a lipogenic enzyme found in the ER, which plays a key role in energy metabolism. It can catalyze the conversion of palmitoyl-CoA and stearoyl-CoA into palmitoyl-CoA and oleoyl and oleoyl-CoA. Palmitoyl-CoA and oleoyl-CoA can be used as substrates to synthesize palmitoleic acid and oleic acid. Palmitoleic acid and oleic acid are the major components of TG, membrane phospholipids, and cholesterol esters (Ozols, 1997; Ntambi et al., 2002; Kato et al., 2006; Sampath and Ntambi, 2014; ALJohani et al., 2017). SCD1 is critical to the improvement of weight gain and IR caused by a high-fat diet in rats and mice (Gutiérrez-Juárez et al., 2017). Insulin sensitivity was increased, and body fat was decreased in SCD1 knockout mice (Ntambi et al., 2002). SREBP-1c is also up-regulated by insulin receptor substrate 1 (IRS-1) to promote fatty acid synthesis and accumulation in hepatocytes. At the same time, IRS-2 down-regulates forkhead box protein A2 (Foxa2), a positive regulator of fatty acid oxidation. Foxa2 could be activated by the inhibition of IRS-2 to promote fatty acid oxidation in NAFLD patients (Kohjima et al., 2008).

The PI3K/AKT signaling pathway also regulates lipid metabolism and insulin signal transduction (Huang et al., 2014; Abood et al., 2018; Liao et al., 2018; Pan et al., 2018). The expression of PI3K and AKT proteins in the liver of NAFLD rats was significantly lower than those of normal rats (Liu et al., 2012; Matsuda et al., 2013). The p85 subunit of PI3K can bind to IRS and anchor in the cell membrane, and then activate the p110 subunit to regulate the transduction of glucose uptake signal in adipocytes and hepatocytes (Foster et al., 2003). AKT inhibits the expression of fatty acid oxidation genes and regulates glucose and lipid metabolism in the liver (Li et al., 2007). PARP-1 is an upstream regulator of PI3K/AKT signaling pathway. Its' over activation can lead to the inhibition of fatty acid β-oxidation (Cantó and Auwerx, 2011). The inhibition of PARP-1 can activate the PI3K/AKT pathway to down-regulate the expression of the fat synthesis gene SREBP-1c, reduce lipid synthesis and accumulation, and improve fatty liver disease in HFHS diet mice (Wang et al., 2019). In addition, inhibition of PARP-1 can also increase NAD^+^ content and SIRT1 activity, enhance lipid metabolism, and improve hepatic steatosis in mice fed a high-fat diet (Kraus and Hottiger, 2013). Meanwhile, the PI3K/AKT signaling pathway also plays an important regulatory role in AFLD. Ethanol intake produces excessive ROS. The imbalance of ROS can lead to cellular oxidative stress. Oxidative stress down-regulates the expression of PI3K/AKT, and then inhibits the expression of its downstream target Nrf2 (Liu et al., 2019). The inhibition of Nrf2 down-regulates the expression of PPARγ (Lee, 2017). PPARγ can reduce the secretion of pro-inflammatory factors by inhibiting the activation of NLRP3 inflammasomes, which then interfere with lipid metabolism. NLRP3 inflammasomes are critical for the initiation of liver inflammation and the progression from hepatic steatosis to NASH. The inhibition of NLRP3 inflammasomes improves the inflammation and oxidative stress levels in AFLD mice (Haneklaus and O'Neill, 2015; Hughes and O'Neill, 2018; Meng et al., 2019).

## Therapies of NAFLD and AFLD

### Exercise Intervention and Abstinence

Aerobic exercise has been suggested as a potential non-clinical strategy to improve NAFLD. Eight-weeks of aerobic training could alleviate the hepatic steatosis of NAFLD mice by activating AMPK-PPAR-α signaling pathway in obese mice (Diniz et al., 2020). Irisin is derived from mouse skeletal muscles in response to exercises. It decreased body weight and fat mass of diet-induced obesity mice in a dose dependent manner. This effector reduced the concentrations of blood glucose and blood lipid, and eventually reversed hepatic steatosis (Tarantino et al., 2019). In patients with AFLD, the clinical trials showed that abstinence may reverse hepatic steatohepatitis and fibrosis, achieve compensation for liver cirrhosis, and improve survival rate of patients with advanced stage (Pár and Pár, 2019). And NAFLD patients, even if alcohol intake within safe threshold, were still at higher risk of liver disease progression. NAFLD patients should also maintain long-term abstinence (Petroni et al., 2019).

## Clinical Treatment

The antioxidant treatment in NAFLD is still controversial. A short-term study of vitamin E supplementation in obese mice induced by high fat diet suggested that the concentration of ROS should be maintained at a reasonable level *in vivo*, rather than excessive or premature elimination, which can maintain the balance of intracellular energy metabolism, lipid metabolism, and insulin signal transduction (Alcala et al., 2017). There is a certain correlation between vitamin D deficiency and NAFLD. However, the effect of vitamin D was limited in clinical trials. In a systematic analysis of six trials, only two trials showed improved results, and the biomarkers of oxidative stress and inflammation decreased in three trials (Sharifi and Amani, 2019). In a vitamin D supplementation trial of 50 patients with metabolic syndromes, there were no significant differences in TG, high-density lipoprotein (HDL) cholesterol, low-density lipoprotein (LDL) cholesterol, and fasting glucose between experimental and control patients (Makariou et al., 2017). Statins reduced the expression of Perilipin 5 (Plin5) through regulating liver lipid metabolism. Atorvastatin reduced the concentration of TG in the liver by up-regulating PKA-mediated phosphorylation of Plin5 (Gao et al., 2017). Aspirin can improve the levels of serum TG, HDL cholesterol and alanine aminotransferase (ALT) in rabbits fed a cholesterol diet. Aspirin enhanced lipolysis and inhibited lipid synthesis and inflammation by activating PPARδ-AMPK-PGC-1α in HepG2 cells and vascular endothelial cells (Han et al., 2020). The glucagon-like peptide-1 (GLP-1) analog/receptor agonists is a class of drugs used for the treatment of adult T2DM, including exenatide, lixisenatide, liraglutide (LRG), albiglutide, dulaglutide, and semaglutide. Although metformin is still the first-line therapy for the treatment of T2DM, and it also has favorable effects on inflammation control in non-obese patients with T2DM and NAFLD (Mitrovic et al., 2021). But the clinical treatment of GLP-1 analogs or receptor agonists may be considered in patients with contraindications or intolerance to metformin, and in patients whose hemoglobin A1c exceeds the target value by more than 1.5% (Collins and Costello, 2020; Tarantino and Balsano, 2020). LRG can alleviate the hepatic lipid accumulation in HFD mice by increasing the phosphorylation of AMPK and ACC and inhibiting the expression of SREBP1. High-dose LRG has been approved by the FDA as a treatment for obesity in overweight patients with comorbidities. Semaglutide is also approved for the treatment of obesity, which is the most potential risk factor of NAFLD. Compared to LRG treatment, semaglutide treatment showed greater mean placebo-corrected weight reductions than LRG in participant population (12.4 vs. 4.5%). In addition, the dose and frequency of semaglutide injection was lower and the effect was persisted longer than LRG. However, the subject populations above were not the same between the two, which reduced the comparability of the two molecular drugs (He et al., 2016; Hao et al., 2019; Newsome et al., 2021; Wilding et al., 2021).

## Potential Effectors of NAFLD and AFLD

Natural compounds extracted from plants are the main sources for screening bioactive substances that are beneficial to the prevention and treatment of human diseases. Flavonoids are a group of natural substances with different phenolic structures, which are found in many fruits, vegetables, grains, and medicinal plants. Many of these natural extracts have been shown to improve hepatic lipid metabolism and inhibit the occurrence and development of NAFLD and AFLD (Zhang et al., 2013). Here, we discuss some representative natural products with potential therapeutic effects on NAFLD and AFLD (Table 1).

**Table 1:** Potential effectors shown to protect the liver from NAFLD and AFLD.

	**Effectors**	**Treatment**	**Experimental model**
NAFLD	Fisetin	20 mg/kg bw/twice a week for 10 weeks	HFD-induced NAFLD mice
	Apple polyphenol extract (APE)	20 μg/ml APE for 24 h	HepG2 cells
	Vine tea polyphenol (VTP)	0.5, 1, and 2% VTP for 12 weeks	Male C57BL/6N mice fed a western diet
	Oleuropein (Ole)	3% Ole for 16 weeks	HFD C57BL/6J mice
	Apigenin (API)	30 mg/kg bw/daily intraperitoneal injection for 3 weeks	Male C57BL/6J mice
	Puerarin	100 and 200 mg/kg bw/daily intraperitoneal injection for 4 weeks	Male C57BL/6J mice
	Curcumin	50 and 100 mg/kg bw/daily by oral gavage for 4 weeks	C57BL/6 mice fed high-fat and high-fructose diet
		250 mg/day for 8 weeks	NAFLD patients
	Rosmarinic acid (RA)	200 mg/kg/day for 10 weeks	Male SD rats fed a high fat diet
		5, 10, 20 μg/ml for 24 h	L02 cells with oleic acid
AFLD	Magnolol	5, 10, and 20 mg/kg BW/day intraperitoneal injection for 3 days	Male BALB/c mice
	Dihydromyricetin (DMY)	75 and 150 mg/kg bw/day for 6 weeks	Male C57BL/6 mice
	Gentianae macrophyllae root extract (GME)	20, 40, and 100 mg/kg bw/day gastric gavage for 19 days	Adult male and female Kunming mice
	Blueberry polyphenol	100 and 200 mg/kg bw/day for 1 month	Male C57BL/6J
	Taxifolin (TAX)	1, 5, and 25 mg/kg bw/day for 4 weeks	Male ICR mice
		25, 50, 100, and 200 μM for 1 h	HepG2 cells

## NAFLD Effectors

Flavonoids have been shown to improve NAFLD in short-term randomized trials and animal studies. In a prospective study involving 2,694 adults, the intake of flavonoid-rich foods was gradually correlated with a decrease in the risk of NAFLD progression mediated by reductions in serum cholesterol and HOMA-IR (Zhong et al., 2021). Fisetin can activate the SIRT1/AMPK pathway to decrease lipid accumulation and increase fatty acid β-oxidation in hepatocytes (Liou et al., 2018). Apple polyphenol extract (APE) could activate SIRT1/AMPK pathway to regulate the autophagy in HepG2 cells, which alleviated the free-fatty-acid-induced lipid accumulation (Li et al., 2021). Vine tea polyphenol (VTP) extracted from Chinese herb *Ampelopsis grossedentata* reduced serum cholesterol and TG levels and decreased hepatic lipid accumulation in male C57BL/6N mice fed a western diet (WD). VTP inhibits lipogenesis by activating phosphorylation of AMPKα and PPARα and reducing the levels of SREBP1 and FAS. At the same time, it was found that VTP can also activate Nrf2-mediated HO-1 and quinone oxidoreductase (NQO1) expression and reduce liver thiobarbituric acid reactive substance (TBARS) levels to prevent liver oxidative stress. Dihydromyricetin (DMY), which is rich in VTP, is a potential bioactive compound to prevent NAFLD (Xie et al., 2020). Oleuropein (Ole) can activate autophagy to improve hepatic steatosis in mice. Ole was able to up-regulate the phosphorylation of Unc-51-like-kinase1 (ULK1) depend on AMPK to induce the autophagy (Porcu et al., 2018). Apigenin (API), a peroxisomal proliferator-activated receptor γ modulator (PPARM), improves the lipid accumulation and oxidative stress of NAFLD by regulating Nrf2 and PPARγ in mice (Panda and Kar, 2007; Tong and Pelling, 2013; Feng et al., 2017). Puerarin is a bioactive isoflavone compound isolated from puerarin root. Puerarin can protect against hepatic steatosis, inflammation, and fibrosis, and improve NAFLD by regulating PARP-1/PI3K/AKT signaling pathway in male C57BL/6J mice (Wang S. et al., 2016). Curcumin is a kind of natural polyphenol, which improves anti-inflammation, anti-oxidation, and lipid metabolism (Um et al., 2013; Wang L. et al., 2016). In a randomized controlled trial of 45 patients with NAFLD, curcumin significantly reduced body weight, waist circumference, body fat percent, and body mass index (BMI) comparing with the placebo group (Mirhafez et al., 2021). Curcumin inhibits hepatic lipid synthesis and promotes bile acid metabolism through NRF2 /FXR/LXRα pathway, and effectively improves NAFLD induced by high-fat and high-fructose diet in C57BL/6 mice (Yan et al., 2018). Rosmarinic acid (RA) is a phenolic compound, which improves NAFLD through by down-regulating TAZ both in male SD rats fed a high fat diet and L02 cells stimulated with oleic acid (Luo et al., 2021).

## AFLD Effectors

Magnolol is a phenolic active substance extracted from the traditional Chinese medicine *Magnolia officinalis*. Magnolol can inhibit oxidative stress by up-regulating the phosphorylation of PI3K and AKT and increasing the expression of downstream Nrf2, HO-1, and PPARγ in male BALB/c mice. Magnolol can also inhibit the activation of NLRP3 inflammasome to reduce the inflammatory response. Magnolol may activate PI3K/AKT/NRF2/PPARγ pathway and inhibit the NLRP3 inflammasome to prevent and improve AFLD in mice (Liu et al., 2019). Dihydromyricetin (DMY) is a kind of flavonoid extracted from *Ampelopsis grossedentata*. DMY has the effects of anti-oxidation, anti-inflammation, anti-bacterial, and improving IR of skeletal muscle (Shen et al., 2012; Jiang et al., 2014; Shi et al., 2015). For ethanol-induced liver injury, DMY can promote the activation of Nrf2 and efficiently induce Keap-1 degradation by regulating p62. DMY can reduce hepatic steatosis and inflammation response in the pathological process of AFLD in male C57BL/6 mice (Qiu et al., 2017). The *Gentianae macrophyllae* root extract (GME) was the main source of gentiopicroside and swertiamarin (Huang R. et al., 2020). These two secoiridoid compounds have been shown to prevent and improve cellular inflammatory responses in rats (Saravanan et al., 2014; Zhao et al., 2015). In ethanol-induced AFLD mice, GME significantly inhibited the expression of pro-inflammatory cytokines TNF- α, IL-1, and IL-6 by inhibiting the phosphorylation of JNK and P38 (Cui et al., 2019). Moreover, the ameliorative effect of GME on AFLD was achieved in a dose-dependent manner. In ethanol-induced AFLD C57BL/6J mice, the expression of fat synthesis-related genes SREBP1, FAS and ACCα were increased, and the expression of lipolytic genes ATGL and Sirt1 were decreased. Blueberry polyphenols significantly reverse this process by promoting hepatocyte autophagy, accelerating lipolysis, and reducing lipid accumulation, but it did not change the expression of these genes in healthy control mice (Zhuge et al., 2020). Excessive alcohol consumption and a high-fat diet (HFD) can promote steatohepatitis, leading to comorbidities of NAFLD and AFLD. An *in vivo* model was established by HFD with a single dose of ethanol in male ICR mice, and an *in vitro* model was established by inducing lipid accumulation in HepG2 cells with oleic acid or palmitic acid. Taxifolin (TAX) is a dihydroxyflavonoid compound that regulated lipid synthesis by inhibiting the expression of SREBP1 and up-regulating the level of PPARγ. In addition, TAX also inhibited the inflammatory response caused by caspase-1 activation. TAX showed a potential therapeutic effect on NAFLD and AFLD caused by ethanol combined with HFD-induced steatohepatitis (Zhan et al., 2021).

## Probiotics/Prebiotics

The gut microbiota played an important role in host metabolism, nutrient absorption, energy utilization, and immune function (Tarantino et al., 2019). The disruption of commensal homeostasis of gut microbiota-host may generate a series of chronic metabolism diseases, such as obesity, T2DM, and NAFLD (Zhang P. et al., 2018). The ecological imbalance of gut microbiota can stimulate liver fat storage by regulating intestinal permeability, dietary choline metabolism, bile acid metabolism, and the production of mild inflammation and endogenous ethanol (Arslan, 2014). NAFLD patients showed an overgrowth of gut microbiota, increased intestinal permeability and paracellular leakage of intestinal lumen antigens, which promotned the development of NASH. Increased *Proteobacteria, E. coli*, and *Negativicutes*, and decreased *Firmicutes* were observed in the intestines of patients with advanced fibrosis and NAFLD (Arslan, 2014). NAFLD mice were treated with *Lactobacillus bulgaricus, Lactobacillus casei, Lactobacillus helveticus*, and *Pediococcus* Kid7, either alone or in combination. The results demonstrated that probiotics had a positive effect on NAFLD by regulating gut microbiota, interfering with inflammatory response, tumor necrosis factor α (TNFα), and interleukin 1β (IL-1β) (Lee et al., 2020). The increase in the proportion of lactic acid bacteria in the feces of NAFLD mice is closely related to the reduction of intestinal inflammation and the increase of GLP-1 concentration. GLP-1 affects obesity by delaying gastric emptying decreasing food intake, and modulates glucose homeostasis by stimulating insulin secretion. A meta-analysis showed that probiotic therapies can improve NAFLD by reducing AST, ALT, total cholesterol, TNF-α, and IR in four randomized trials involving 134 NAFLD and NASH patients (Kanda et al., 2020). Because obesity is one of the main driving factors of NAFLD, in the study of probiotics to improve NAFLD, it is necessary to specifically evaluate the changes of body fat rate, TG, insulin sensitivity, fat inflammation, and various metabolic parameters to determine its function (Ahn et al., 2019). In a study of lean donor fecal microbiota transplantation (FMT), although the experiment proved to be safe, it did not reduce the BMI of patients with obesity and other metabolic diseases. Therefore, the strategy of treating chronic metabolic diseases with probiotics still needs to be carefully evaluated (Zhang et al., 2019). VTP was able to increase the relative abundance of intestinal *Akkermansia* (*AKK*) and decrease the ratio of *Firmicutes/Bacteroidetes* in mice fed a WD. VTP may prevent WD-induced NAFLD by balancing lipid metabolism, liver oxidative stress and gut microbiota (Xie et al., 2020). In addition, compared to healthy people, the decreased abundance of *AKK* in the gut microbiota of alcoholic hepatitis patients was positively correlated with the severity of AFLD. *AKK* can increase the thickness of intestinal mucus and the expression of tight junction protein to prevent alcohol-induced gastro-intestinal leakage. The increased abundance of *AKK* can improve the neutrophil infiltration and liver injury in AFLD patients. Exogenous supplementation of *AKK* or promoting the proliferation of *AKK* through the diet may be a potential strategy for the treatment of AFLD (Grander et al., 2018).

## Conclusion

NAFLD and AFLD are the most prevalent metabolic liver diseases worldwide. Their complex pathogenesis restricts the progress of prevention, diagnosis, and treatment. The prevalence of NAFLD in China (22.4%) is similar to that in the United States (24.13%) and Europe (23.71%), with an estimated 173–338 million people affected by NAFLD. The prevalence of ALD in China is 4.5%, slightly lower than that in the United States (6.2%) and Europe (6%), and higher than that in Japan (1.56–2.34%), with 62 million people that are affected by AFLD. The high prevalence of metabolic liver diseases such as NAFLD and ALD has provoked concern (Xiao et al., 2019). In order to prevent the rapid rise of the incidence of metabolic liver disease, we urgently need safe and effective drug intervention and treatment. Major economies in the world have invested a lot of research funds to promote high-quality basic/clinical research and transformation. Currently, despite hundreds of promising pharmacological trials in progress, no registered drugs have been approved for the treatment of metabolic liver diseases, including NAFLD and AFLD. From 2009 to 2017, none of the new drugs approved by the CFDA were for metabolic liver disease. During the same period, 59 new drugs for liver diseases were approved by the United States Food and Drug Administration (FDA, USA), the European Medicines Agency (EMA, EU), and the Medicines and Medical Devices Agency (PMDA, Japan), almost all of which were for the treatment of viral hepatitis and cirrhosis.

**Figure 1:**
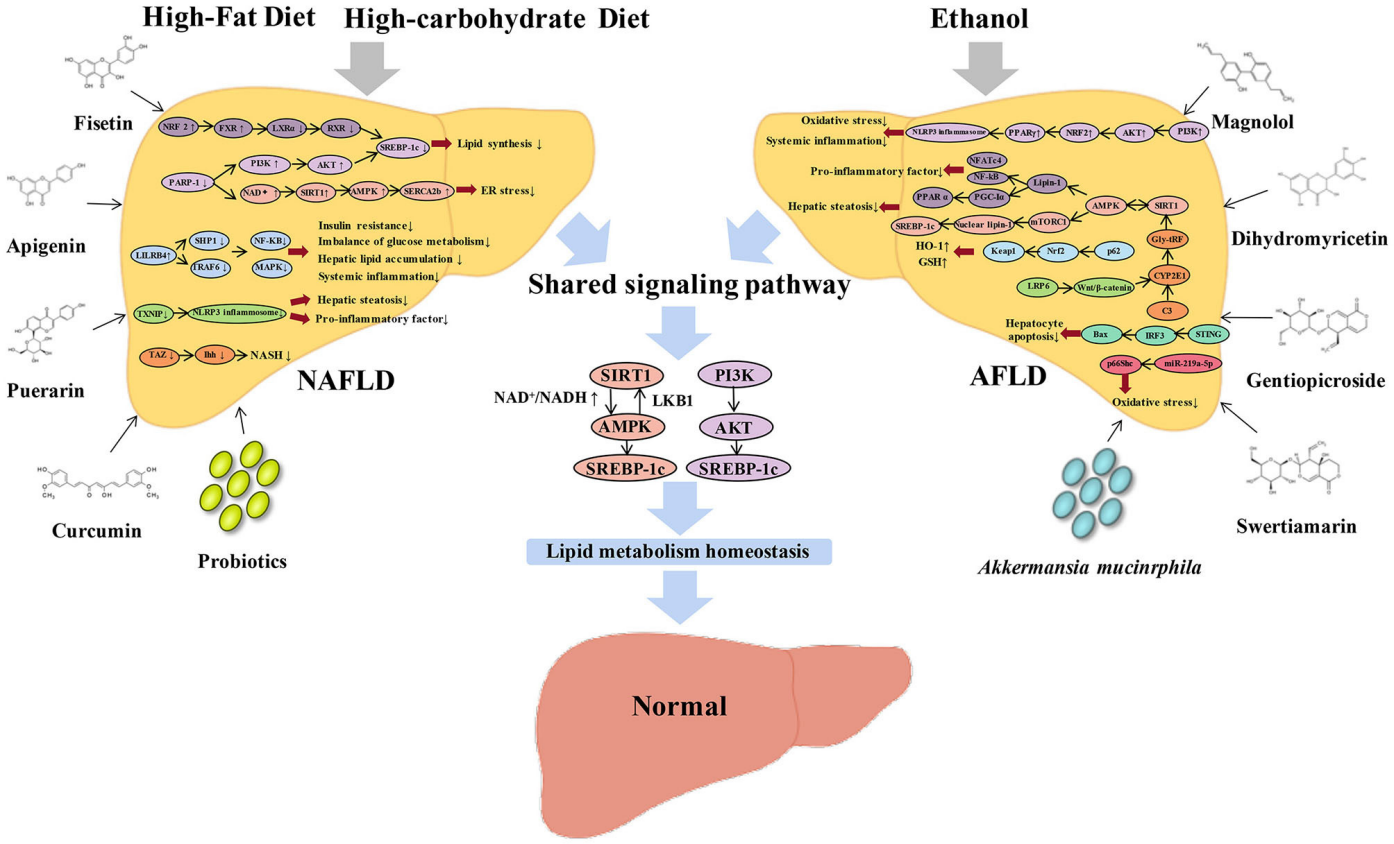
Graphical representation of the comparative review of NAFLD and AFLD signaling pathway and effectors. High fat and high carbohydrate diet-induced NAFLD can be modulated by Nrf2/FXR/LXRα/RXR/SREBP-1c, PI3K/AKT/SREBP-1c, AMPK/SERCA2b, LILRB4/SHP1/TRAF6/NF-κB/MAPK, and TAZ/Ihh signaling pathways. Ethanol-induced AFLD can be modulated by STING-IRF3-Bax, C3 /CYP2E1/Gly-tRF/SIRT1, LRP6/Wnt/β-catenin/CYP2E1, and miR-219a-5p/p66Shc signaling pathways. NAFLD and AFLD share two signaling pathways, SIRT1/AMPK/SREBP-1c and PI3K/AKT/SREBP-1c, while other pathways are owned by each other specifically. A variety of natural flavonoids can improve NAFLD, including fisetin, apigenin, puerarin, and curcumin; and AFLD, including magnolol, dihydromyricetin, gentiopicroside, and swertiamarin. In addition, NAFLD and AFLD can also be improved by probiotics and *Akkermansia muciniphila*. Ultimately, NAFLD and AFLD could be reversed to achieve a homeostasis of lipid metabolism.

It has been suggested that NAFLD is an early physiological adaptation of human liver under adverse living conditions, and it is only a physiological response (Tarantino et al., 2019). However, it has caused more serious pathological metabolic diseases in the modern lifestyle (Muñoz-Garach et al., 2016). A comprehensive understanding of NAFLD and AFLD signaling pathways and clinical treatment will help identify biomarkers and therapeutic targets for early detection and management. Although NAFLD and AFLD are major challenging public health problems, they are preventable. In daily life, lifestyle changes are still the first-line treatment for NAFLD and AFLD. Dietary ingredients and exercises that reduce the risk of NALFD can effectively reverse early fatty liver disease. AFLD can also be reversed by stopping alcohol intake and changing lifestyle. The current basic/clinical studies on NAFLD and AFLD are mainly focused on: ① Anti-inflammatory, anti-oxidant, and anti-fibrotic treatments; ② Improve insulin resistance; ③ Improve lipid metabolism and increase fatty acid β-oxidation; ④ Reduce oxidative stress, hepatic gluconeogenesis, lipid synthesis, and hepatic steatosis; ⑤ Activation and inhibitors of key targets in the glucose and lipid metabolism pathway. By comparing the specific and shared molecular regulatory mechanisms, clinical treatments, and potential effectors of NAFLD and AFLD, we provide mutual reference strategies for the prevention and treatment of NAFLD, AFLD, and their related metabolic diseases (Figure 1). Furthermore, it provides enlightenment and ideas for the discovery and development of safe and effective drugs in the field of metabolic liver disease.

## Author Contributions

PZ and XM contributed to the conception, design, and manuscript writing. WW, RG, MM, WS, DL, RC, BS, and XT contributed substantially to the writing and revision of the manuscript and approved its final version. All authors contributed to the article and approved the submitted version.

## Conflict of Interest

The authors declare that the research was conducted in the absence of any commercial or financial relationships that could be construed as a potential conflict of interest.

## Publisher's Note

All claims expressed in this article are solely those of the authors and do not necessarily represent those of their affiliated organizations, or those of the publisher, the editors and the reviewers. Any product that may be evaluated in this article, or claim that may be made by its manufacturer, is not guaranteed or endorsed by the publisher.
